# Changes in the Volatile Components of Candied Kumquats in Different Processing Methodologies with Headspace–Gas Chromatography–Ion Mobility Spectrometry

**DOI:** 10.3390/molecules24173053

**Published:** 2019-08-22

**Authors:** Xiao Hu, Rongrong Wang, Jiajing Guo, Keda Ge, Gaoyang Li, Fuhua Fu, Shenghua Ding, Yang Shan

**Affiliations:** 1 Longping Branch Graduate School, Hunan University, Changsha 410125, China; 2 Provincial Key Laboratory for Fruits and Vegetables Storage Processing and Quality Safety, Agricultural Product Processing Institute, Hunan Academy of Agricultural Sciences, Changsha 410125, China; 3 College of Food Science and Technology, Hunan Agricultural University, Changsha 410128, China

**Keywords:** preserved fruit processing, candied kumquats, sugar osmosis, hot-air drying, volatile components, HS–GC–IMS, PCA

## Abstract

The effects of two different processing methods on the volatile components of candied kumquats were investigated via headspace–gas chromatography–ion mobility spectrometry (HS–GC–IMS). The characteristic volatile fingerprints of fresh kumquats (FKs), vacuum sugaring osmosis combined with hot-air drying kumquats (VS-ADKs), and atmospheric pressure sugaring osmosis combined with hot-air drying kumquats (AS-ADKs) were established using 3D topographic plots. From the fingerprints, 40 signal peaks for 22 compounds were confirmed and quantified in all types of kumquats, namely, two terpenes, four esters, seven aldehydes, three ketones, and six alcohols. 3-Pentanone was identified as the major component of FKs; followed by 1-hexanol and the *Z*-3-hexen-1-ol dimer. The hexanal dimer, 2-hexen-1-ol, and the ethyl acetate dimer were the major markers of VS-ADKs. Benzaldehyde and furfurol were the prominent constituent parts of AS-ADKs. Compared with that in FKs, the pentanal and dimethyl ketone contents of VS-ADKs and AS-ADKs exhibited a dramatic increase (*p* < 0.05). By contrast, the change in ethanol dimer tended to decrease (*p* < 0.05). Principal component analysis (PCA) clearly showed that the samples, which were distributed in a separate space could be well-distinguished. Furthermore, the similarity of different processed kumquats and their corresponding volatile components was demonstrated via heat map clustering analysis. The results confirmed the potential of HS–GC–IMS-based approaches to evaluate processed kumquats with various volatile profiles.

## 1. Introduction

Kumquat plants (*Citrus japonica*), which belong to the Rutaceae family, are native to South Asia and Asia-Pacific. They have long been cultivated in China, Japan, the Philippines, and Southeast Asia [1]. Kumquats bear small and elliptical-shaped fruits with a smooth, bright orange rind. The fruits are typically eaten raw as a whole, including the peel, but excluding the seeds. They are nutritious because they are excellent sources of phytochemicals, such as carotenoids, flavonoids, phenolic compounds, minerals, and vitamins [2]. The antioxidant, antitumor, antimicrobial, and anti-inflammatory activities of kumquat have been summarized by Lou et al. [3]. Kumquats can be used to produce liqueurs, marmalades, and sauces, and they can also be candied or preserved whole in sugar syrup [4]. Kumquats are typically preserved because fresh kumquats (FKs), which have a high moisture content, easily become perishable and deteriorate if they are not handled properly. Kumquats are also candied to prolong their storage time and improve their acceptability by conferring them with pleasant flavors. As a kind of traditional Chinese food, preserved kumquat fruit variety with a special taste and flavor is known as the homology of medicine and food. It can not only be eaten directly and added to other foods to improve flavor, but also shows therapeutic effects, including being appetizing, enhancing cold resistance, and treating a cough [5]. The characterization of preserved kumquat fruit is helpful to guide its practical production and commercialization. Its processing method combines the effects of sugar impregnation with drying and depends on the osmotic dehydration of sugar to achieve a preservation effect [6]. Osmosis with sugar at atmospheric pressure is a conventional method of preserving fruits in China. It is a relatively inefficient and time-consuming process [7]. Vacuum osmosis with sugar is a method by which the gas in the fruit tissues is extracted by a vacuum. The diffusion channels of sugar solution are opened to promote the rapid osmosis of sugar solution and shorten the production cycle [8]. Subsequently, fruits are dried to effectively control their moisture content and inhibit the growth of the microorganism [6]. Simultaneously, drying can confer products with unique olfactory and organoleptic properties. Therefore, sugar osmosis and drying are two key operations employed to determine the quality and shelf life of preserved kumquat fruit.

The flavor of food is crucial for determining its overall unique sensory characteristics and evaluating its nutritional value and freshness [9]. Flavor and aroma also play important roles in the consumer acceptance of fruits. These characteristics are attributed to different chemical compounds (e.g., esters, terpenes, alcohols, and aldehydes) [10,11]. Previous studies have focused on the aroma of FK’s peel oil using different extraction methods [12,13], the measurement of major volatile compounds in different citrus species [14,15], and the accumulation and variation of volatile components during the fruit development of kumquat [16]. These studies proved that terpenes are the major compounds, while limonene is the most abundant compound, in kumquats. They also determined that the essential oils of kumquat are mostly composed of volatile monoterpenoids and sesquiterpenoids [17]; Moreover, their oxygenated derivatives, aldehydes, alcohols, and esters accounted for approximately 85%–99% of the oils [18]. At present, however, information about the changes in volatile components of the pulps of preserved kumquat fruits during processing remains unavailable. This experiment studied the flavor variations of preserved kumquat fruit during processing, which could monitor the different flavor compositions at different stages and infer the change mechanism of flavors. Flavor changes can also be used to judge and measure the quality of preserved kumquat fruit differences. The operating points of the processing can be summarized and optimized to meet the tastes and preferences of different consumers.

Ion mobility spectrometry (IMS) was developed in the late 1960s for the rapid detection of drugs, explosives, and chemical agents [19]. When conducting IMS, the samples being tested are gasified by an ion source and turned into gas molecules. Then, the gas molecules are chemically ionized with a certain amount of electric charge and moved under the action of an electric field to create an ion map that changes with time. IMS is a convenient and efficient method that exhibits simple sample preparation, a short incubation period, a quick analytical time, and a superior sensitivity. With the right sampling approach, IMS enables the detection of a large number of compounds from different chemical families, such as alcohols, aldehydes, aromatics, esters, and ketones, and even from the most complicated and problematic matrices, such as food and agricultural products [20]. Combining IMS with other instruments is a suitable and effective method for maximizing its advantages and producing good analysis results. In recent years, headspace–gas chromatography–IMS (HS–GC–IMS) has been extensively applied to investigate volatile compounds, such as virgin olive oil [21], wine [22], eggs [23], Iberian ham [24], honey [25], goat cheeses [26], *Tricholoma matsutake* Sing. [27,28], Chinese material medica [29], and white bread [30]. HS–GC–IMS is used in oil identification, wine classification, product (e.g., egg, fish, and meat) freshness detection, fermentation product monitoring, and target compound quantitative analysis. However, the flavor formation of kumquat is a relatively complex process, and few research studies have adopted HS–GC–IMS to monitor this process. Therefore, HS–GC–IMS can be used to establish the fingerprints of volatile compounds in candied kumquats.

Principal component analysis (PCA) was used as a non-supervised multivariate data analysis technique for dimensionality reduction and numerical classification [31]. Garrido-Delgado et al. [22] analyzed the 36 peaks from a chromatogram and achieved a good classification of four types of wine samples via PCA. The goat cheeses manufactured with raw or pasteurized milk displayed separation and showed two clusters via PCA in the research of Gallegos et al. [26]. Additionally, the PCA results clearly showed that different fresh and dried *T. matsutake* samples in a relatively independent space would be well-distinguished [27]. The different varieties of fresh *Chrysanthemum morifolium* flowers were differentiated and classified by fingerprint similarity evaluation and PCA [32]. However, little research has been performed on the classification of candied kumquats with different processing methodologies based on volatile compounds.

In the present study, the effects of atmospheric and vacuum osmosis with sugar on the flavor substances of dried fruits and flavor changes in preserved kumquats with an extended drying time were analyzed. Several target compounds in the samples were detected using HS–GC–IMS, PCA, and a heat map. The established fingerprints can be used to benchmark key analytes for controlling the production chain of preserved kumquats and provide enhanced flavor quality. This work may offer a novel method for identifying good flavor in preserved kumquats and predicting an optimal processing time.

## 2. Results and Discussion

### 2.1. HS–GC–IMS Topographic Plots of FKs, VS-ADKs, and AS-ADKs

**Figure 1:**
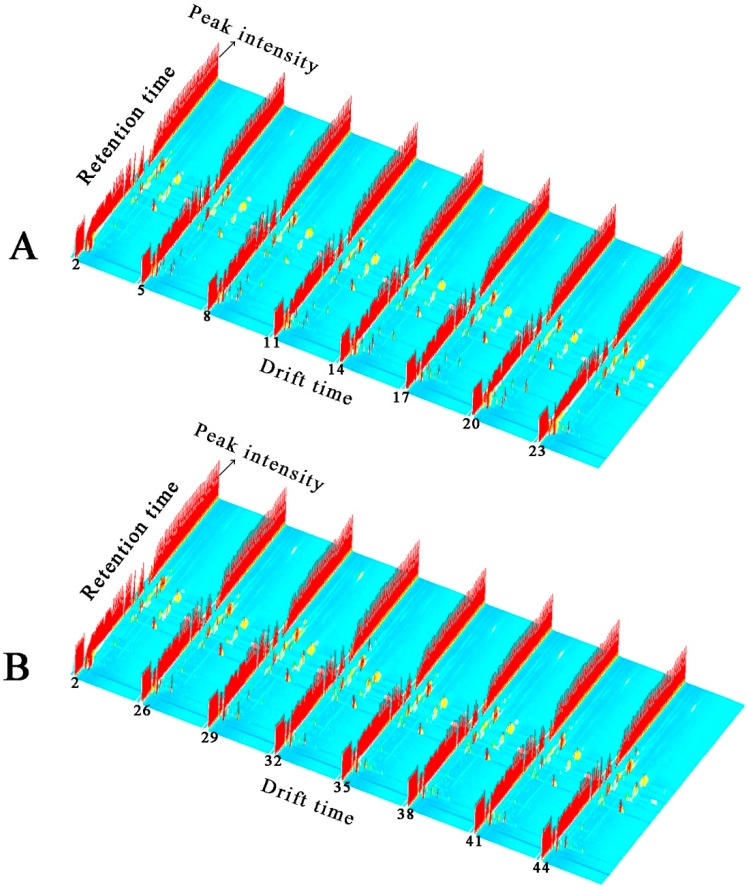
3D topographic images of fresh kumquats (FKs) and vacuum osmosis with sugar and then hot air drying kumquats (VS-ADKs). 2: FKs; 5: kumquats with vacuum sugaring and then hot air drying for 0 h (VS-ADKs for 0 h); 8: VS-ADKs for 1 h; 11: VS-ADKs for 2 h; 14: VS-ADKs for 4 h; 17: VS-ADKs for 6 h; 20: VS-ADKs for 8 h; 23: VS-ADKs for 10 h; These are showed in (**A**). 3D topographic images of fresh kumquats (FKs) and atmospheric pressure osmosis with sugar and then hot air drying kumquats (AS-ADKs). 2: FKs; 26: kumquats with atmospheric pressure sugaring and then hot air drying for 0 h (AS-ADKs for 0 h); 29: AS-ADKs for 1 h; 32: AS-ADKs for 2 h; 35: AS-ADKs for 4 h; 38: AS-ADKs for 6 h; 41: AS-ADKs for 8 h; and 44: AS-ADKs for 10 h; These are showed in (**B**).

The volatile components of FKs and the preserved kumquats with different processing methodologies (i.e., different osmosis and drying methods) were obtained via HS–GC–IMS analysis. A Flavor Spec^®^ instrument was used to generate data in the form of a 3D spectrum, as shown in Figure 1. The volatile components in different samples demonstrated varying peak intensities. As shown in Figure 1A,B, several new small peak signals of the volatile components of VS-ADKs (5, 8, 11, 14, 17, 20, and 23) and AS-ADKs (26, 29, 32, 35, 38, 41, and 44) appeared compared with those of FKs (2). The number of new peaks in AS-ADKs (Figure 1B) was less than that in VS-ADKs (Figure 1A). This result may be attributed to the conclusion that osmotic impregnation could enrich several volatile substances, while vacuum impregnation provides higher aroma enhancement than atmospheric pressure [33]. This result may also be positively correlated with the drying process. Yang et al. affirmed that the drying process can significantly promote the generation of alcohols, acids, and esters, which are the major volatile compounds of dried *Flammulina velutipes* [34]. After 10 h of hot-air drying treatment, as shown in samples no. 23 and no. 44, several peak signals disappeared. This result is similar to the phenomenon in which different areas of *T. matsutake* Sing. lost their flavors after hot-air drying at 60 °C. This condition may be due to the degradation of long-chain compounds during thermal drying [27,28].

**Figure 2:**
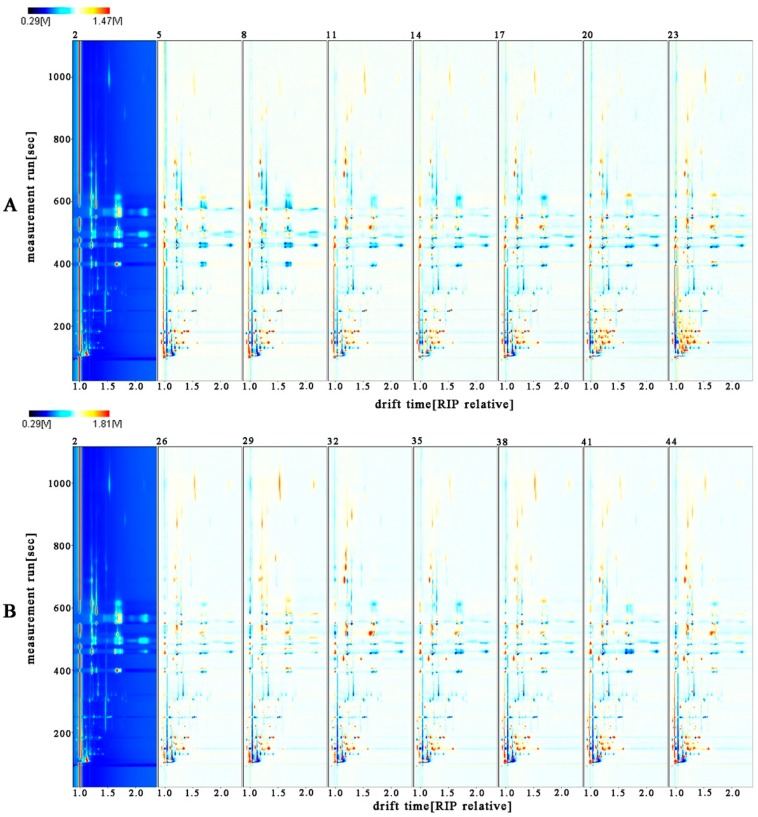
2D topographic images of fresh kumquats (FKs) and vacuum osmosis with sugar and then hot air drying kumquats (VS-ADKs). 2: FKs; 5: kumquats with vacuum sugaring and then hot air drying for 0 h (VS-ADKs for 0 h); 8: VS-ADKs for 1 h; 11: VS-ADKs for 2 h; 14: VS-ADKs for 4 h; 17: VS-ADKs for 6 h; 20: VS-ADKs for 8 h; 23: VS-ADKs for 10 h; These are showed in (**A**). 2D topographic images of fresh kumquats (FKs) and atmospheric pressure osmosis with sugar and then hot air drying kumquats (AS-ADKs). 2: FKs; 26: kumquats with atmospheric pressure sugaring and then hot air drying for 0 h (AS-ADKs for 0 h); 29: AS-ADKs for 1 h; 32: AS-ADKs for 2 h; 35: AS-ADKs for 4 h; 38: AS-ADKs for 6 h; 41: AS-ADKs for 8 h; and 44: AS-ADKs for 10 h; These are showed in (**B**).

The remaining volatile organic compounds (VOCs) in kumquats were difficult to observe. Therefore, the vertical view was used for comparison, as shown in Figure 2. The background of FKs is blue, and the red vertical line at horizontal coordinate 1.0 is the reactive ion peak (RIP, normalized drift time of 7.83). Each point on the right side of RIP symbolizes a VOC. To clearly compare the differences among samples, the differential comparison mode can be adopted. That is, the spectral diagram of one sample (2) was selected as the reference, while the spectral diagram of other samples was deducted as the reference. If two VOCs were identical, then the background after deduction was white. Red spots meant that the concentration of the substance was higher than that of the reference, whereas blue spots meant that the concentration of the substance was lower than that of the reference. The majority of the data were located at the topographic plot zone from 100 s to 800 s of retention time and from 1.0 to 1.5 of drift time.

As the drying time increased under the same control, the red spot areas between 100 s and 200 s of retention time in VS-ADKs were distinctly larger than those in AS-ADKs. Therefore, the concentration of several volatile substances in VS-ADKs was higher than that in AS-ADKs. This result may be attributed to the release of certain components after vacuum impregnation and drying treatment. By contrast, the blue spot areas in VS-ADKs were less than that those in AS-ADKs. Therefore, the concentrations of other volatile substances in AS-ADKs were lower than those in VS-ADKs. This result may be due to the loss of certain components (sensitive to temperature and easily decomposed or degraded) after atmospheric pressure impregnation and drying treatment. These changes may be related to the Maillard reaction. The formation of derived flavor compounds was dependent on the temperature or sugar type [35,36]. Changes in signals in the retention time of 200–800 s were unapparent in Figure 2A,B. On this basis, the information in Figure 2 is consistent with that in Figure 1. The other contrast areas were not evident. Hence, all the signal peaks were selected for fingerprint comparison to determine the specific composition changes in preserved kumquats.

### 2.2. Differences in the Characteristic Volatile Fingerprints of FKs, VS-ADKs, and AS-ADKs

**Figure 3:**
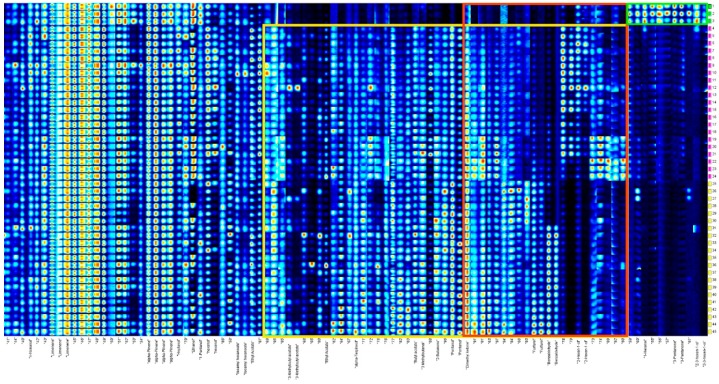
Fingerprints of all the samples generated using the Gallery Plot.

A fingerprint map that involves all plots and areas to compare differences in VOCs among FKs, VS-ADKs, and AS-ADKs for further analysis is shown in Figure 3, where each row represents a sample and each column represents a substance. A total of 40 signal peaks were identified and marked with an existing name. The substances in the green frame, namely, 1-hexanol, 3-pentanone, and *Z*-3-hexene-1-ol, were abundant in FKs, but their content decreased or disappeared after drying. The contents of substances in the yellow frame, namely, isoamyl acetate, ethyl acetate, α-terpineol, butyl acetate, 3-methylbutanal, 2-butanone, dimethyl ketone, pentanal, furfurol, benzaldehyde, and 2-hexene-1-ol, were not found or were extremely low in FKs, but they increased remarkably in VS-ADKs and AS-ADKs. The materials in the red frame, such as dimethyl ketone, furfurol, benzaldehyde, and 2-hexene-1-ol, changed regularly with the extension of the drying time.

**Figure 4:**
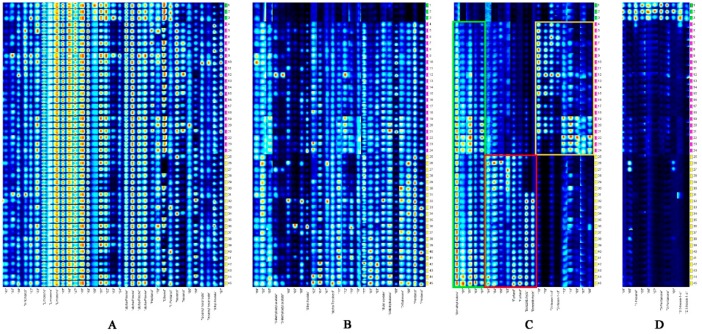
Four parts of the screenshots from the fingerprints. Nine kinds of identified peaks (**A**); Seven kinds of identified peaks (**B**); Four kinds of identified peaks (**C**); Three kinds of identified peaks (**D**).

Given that the spectrum shown in Figure 3 contained too much information for observation, the entire figure was divided into four screenshots and then enlarged and displayed as Figure 4 to obtain the details of the fingerprints. As shown in Figure 4A, nine peaks, including limonene, α-pinene, and ethanol, were confirmed. The brightness variation of the fingerprint of terpenes remained roughly stable. Osmotic treatment activated the decreasing behavior of terpenes in mango [37]. This result is inconsistent with ours. Such inconsistency may be attributed to different fruit matrices, genetics, nutrition, and growth environment [38]. The amount of ethanol in FKs and VS-ADKs showed nearly a similar brightness, but discontinuously existed in AS-ADKs. Hexanal appeared in VS-ADKs, while its fingerprint information in FKs and AS-ADKs was minimal. Ethyl acetate was present in VS-ADKs and AS-ADKs, but its fingerprint in FKs was inferior to that in VS-ADKs. Ethyl acetate tended to augment drastically, probably depending on metabolic pathways related to fermentation during osmosis with sucrose [39]. As shown in Figure 4B, corresponding substances, such as α-terpineol, butyl acetate, 3-methylbutanal, 2-butanone, and pentanal, were only found in VS-ADKs and AS-ADKs. They accounted for the major constituents, while 3-methylbutyl acetate and ethyl acetate accounted for a smaller percentage. As shown in Figure 4C, the substances in the yellow frame, including 2-hexene-1-ol, were the peculiar flavor substances in VS-ADKs. The content of 2-hexene-1-ol gradually decreased with the extension of the drying time. The substances in the red frame, such as furfural and benzaldehyde, could be considered the characteristic flavors of AS-ADKs. The content of furfural was gradually increased as the treatment time increased, while that of benzaldehyde remained basically constant after 2 h of drying. Furans (e.g., furfural) and aldehydes were produced by dehydrating deoxygenated aldose and ketose and degrading Strecker of amino acids in the Maillard reaction, respectively [40]. In the green frame, the concentration of dimethyl ketone in AS-ADKs was higher than that in VS-ADKs. 1-Hexanol, 3-pentanone, and *Z*-3-hexene-1-ol were only detected in FKs, but they hardly existed after drying, as shown in Figure 4D.

### 2.3. Identification of Volatile Substances in FKs, VS-ADKs, and AS-ADKs

**Figure 5:**
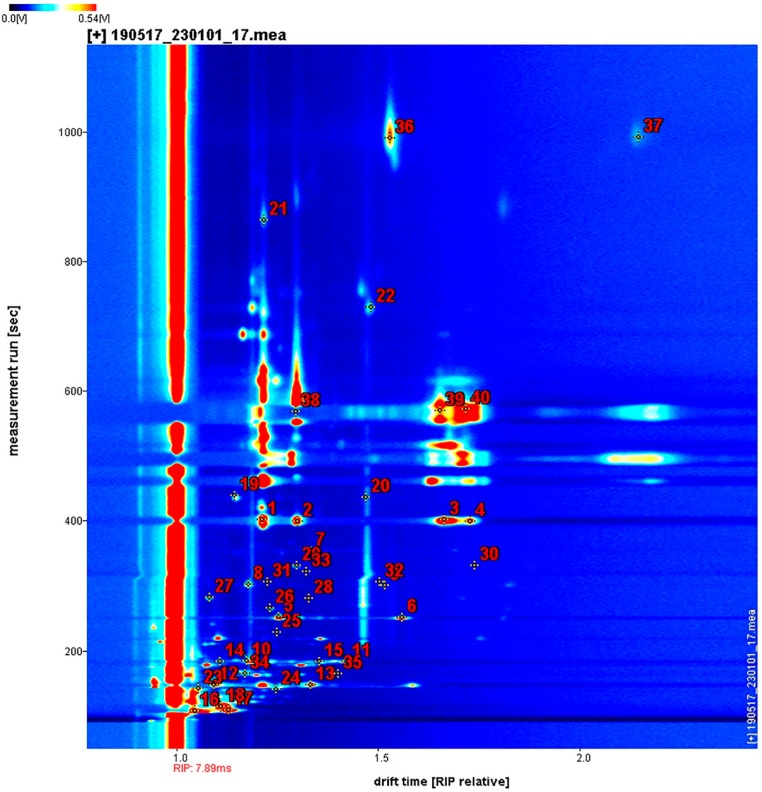
Imaging of volatile compounds represented by HS–GC–IMS spectra with the selected markers obtained from different kumquat samples.

The qualitative analysis of VOCs in FKs was represented by numbers in Figure 5. The imaging of VS-ADKs and AS-ADKs was consistent with that of FKs. A total of 22 typical compounds were identified from the GC×IMS library (Figure 5 and Table 1). Several single compounds may produce multiple signals or spots (e.g., monomers, dimers, and even polymers), which are attributed to their varying concentrations [27]. A single compound was observed with more than one signal during the drift time due to adducts formed between the analyzed ions and neutral molecules (e.g., dimers and trimers) while moving through the drift region [41]. The formation of dimers or high clusters was demonstrated in relation to compounds with a high proton affinity or higher analyte concentration [42]. The monomers of several substances (e.g., 2-butanone, ethanol, and 3-methylbutanal) can be stripped of protons by dimers or interfered with by other substances. Consequently, the correct amount cannot be reflected. These products have similar retention times, but different drift times. Table 1 lists all the identified substances in the samples (40 peaks for 22 compounds), including the compound name, Chemical Abstracts Service (CAS) number, molecular formula, molecular weight, retention index (RI), retention time, and migration time. In summary, VOCs in kumquats include two terpenes, four esters, seven aldehydes, three ketones, and six alcohols.

### 2.4. Distribution and Comparison of Five Types of Volatile Constituents in Different Kumquats

**Figure 6:**
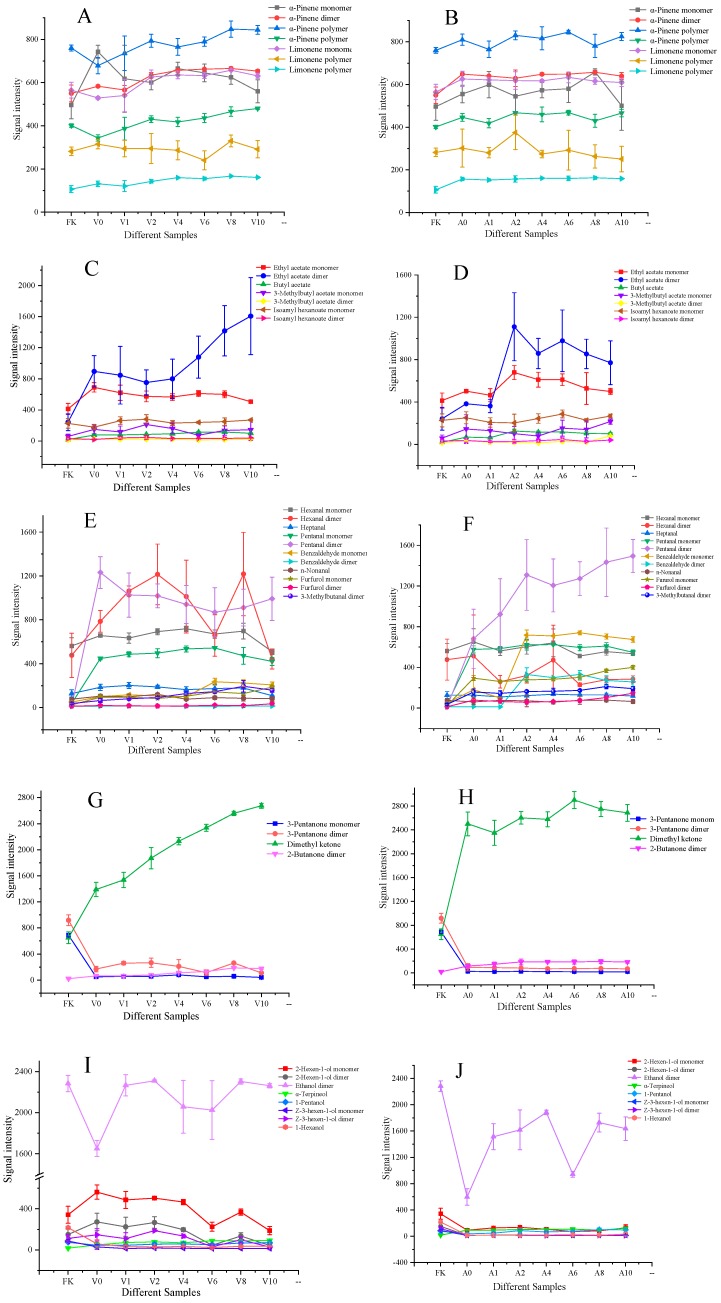
Line graph showing the intensity (mean ± standard deviation) of markers identified in the kumquat samples. Note: FK: FK; V0: VS-ADKs for 0 h; V1: VS-ADKs for 1 h; V2: VS-ADKs for 2 h; V4: VS-ADKs for 4 h; V6: VS-ADKs for 6 h; V8: VS-ADKs for 8 h; V10: VS-ADKs for 10 h. A0: AS-ADKs for 0 h; A1: AS-ADKs for 1 h; A2: AS-ADKs for 2 h; A4: AS-ADKs for 4 h; A6: AS-ADKs for 6 h; A8: AS-ADKs for 8 h; and A10: AS-ADKs for 10 h. (**A**,**B**) Two types of terpenes of FKs, VS-ADKs, and AS-ADKs; (**C**,**D**) Four types of esters of FKs, VS-ADKs, and AS-ADKs are shown; (**E**,**F**) Seven types of aldehydes of FKs, VS-ADKs, and AS-ADKs; (**G**,**H**) Three types of ketones of FKs, VS-ADKs, and AS-ADKs; Six types of alcohols of FKs, (**I**,**J**) VS-ADKs and AS-ADKs.

Using FKs as the reference, five types of volatile components were classified and selected from 22 identified substances to quantitatively distinguish the release of volatile components of kumquats after different processing methods (Figure 6 and Tables S1 and S2). One-way ANOVA was performed to evaluate the differences between categories. Multiple range test–least significant difference was used as a multiple comparison procedure to compare the means. Overall, combined with the exhibition of line charts, the monomers and dimers of 3-pentanone and 1-hexanol may be the markers of FKs. The ethyl acetate dimer, hexanal dimer, pentanal monomer and dimer, dimethyl ketone, and 2-hexen-1-ol monomer and dimer were the major components of VS-ADKs. Pentanal monomers and dimers, benzaldehyde, the furfurol monomer, and dimethyl ketone were the principal components (PCs) of AS-ADKs. Interestingly, all these markers were regarded as important distinguishing markers when samples subjected to periods of vacuum sugaring, atmospheric pressure sugaring, and then hot-air drying were compared.

Two types of terpenes of FKs, VS-ADKs, and AS-ADKs are shown in Figure 6A,B. The content of the α-pinene monomer in V0 was significantly higher than that in FKs (*p* < 0.05) (Tables S1 and S2). However, its content slightly decreased after drying. The figures of the α-pinene dimer and limonene monomer in V2–V10 presented a noticeable increase compared to those in FKs. V10 recorded the highest intensity of α-pinene polymer. Limonene polymers increased. The amounts of α-pinene monomer in A8 and α-pinene dimer in A0–A10 were considerably larger than those in FKs (*p* < 0.05) (Tables S1 and S2). The figures of α-pinene polymers and the limonene monomer and polymers in A0–A10 were on the rise compared with those in FKs. In general, terpenes, particularly α-pinene polymers, exhibited an increasing trend after processing. This finding can be explained by the possible production of terpenes via thermal degradation and the metabolism of carbohydrates and lipids [38]. As a ligand, terpenes are linked with glucose to form glycosidically-derived compounds. Terpenes will be released from glycosyl via thermal catalysis, as evidenced in Thompson seedless raisins in the study of Javed et al. [43].

The detailed differences of the four esters in FKs, VS-ADKs, and AS-ADKs are shown in Figure 6C,D. The normalized signal values of the ethyl acetate monomer increased markedly in V0, while those of the ethyl acetate dimer presented a dramatic increment in V10 (*p* < 0.05) (Tables S1 and S2). The signal values of the ethyl acetate monomer and dimer achieved the highest level in A2. The content of the ethyl acetate dimer in V10 was approximately 1.4 times that in A2. This result may be attributed to the promotion of fermentative ethyl acetate in fruits under anaerobic conditions during vacuum immersion with sugar [37]. Vacuum impregnation reduced the oxygen consumption of tissues more than impregnation performed at atmospheric pressure [44]. Talents et al. proved that the application of vacuum pulse osmosis promoted ester formation in kiwi fruits due to the absence of gas (oxygen) in intercellular spaces [45]. Furthermore, Javed et al. demonstrated that the air-drying method was more effective in producing ethyl acetate via the Embden–Meyerhof–Parnas reaction [43]. The signal intensification of butyl acetate appeared in V1–V10 and A1–A10. Nevertheless, regardless of whether in V1–V10 or A1–A10, the corresponding signals of 3-methylbutyl acetate and isoamyl hexanoate changed indistinctively. Overall, the change in ethyl acetate was the most prominent among all esters.

The variation rule of seven aldehydes varied in the processing treatments (Figure 6E,F). Compared with that in the control group FKs, the signal value of the hexanal monomer increased significantly in V0–V8 (*p* < 0.05) (Tables S1 and S2). The hexanal dimer constituent increased in V0–V4 and V8 to a considerable extent relative to that in FKs. The heptanal component increased slightly in V1–V8. However, no evident changes in hexanal and heptanal components were observed in A0–A10. In V0–V10, the monomer and dimer of pentanal constituents increased sharply (*p* < 0.05) (Tables S1 and S2). In A0–A10, a stepwise increase of the pentanal component was observed with a longer drying time. This result may be related to the oxidation of unsaturated fatty acids (e.g., linoleic and linolenic acids); this process produces a certain amount of fruity flavors via enzymatic breakdown, α-oxidation, and β-oxidation in intact or disrupted fruits [46]. This finding is in accordance with Javed et al. [43], who demonstrated that hexenal, heptenal, and pentanal components in sun-dried and air-dried raisins belong to compounds derived via unsaturated fatty acid oxidation. This finding was also confirmed in the bread study of Pu et al. [30]. In the current experiment, vacuum impregnation and hot-air drying may be favorable to the aforementioned reaction. Apparently, the increment of benzaldehyde in A6 was approximately thrice that in V8 with the extension of the drying time. This result is attributed to the Strecker degradation of isoleucine producing benzaldehyde during the Maillard reaction [47]. The signal value of n-nonanal was essentially constant in V0–V10 and A0–A10. The changes in furfural constituents were similar to those in benzaldehyde. Its content in A0–A10 was also higher than that in V0–V10. The thermal degradation of sugars, such as fructose and glucose, can produce compounds with furans (e.g., furfurols) [48]. On this basis, a conclusion can be drawn that atmospheric pressure impregnation and hot-air drying may be beneficial to the Maillard reaction. Furthermore, the 3-methylbutanal dimer gradually increased in V0–V8 and A0–A8. 3-Methylbutanal can be formed via the Strecker degradation of leucine during the Maillard reaction [49,50]. In general, changes in the hexanal dimer and pentanal were the most prominent in VS-ADKs. By contrast, changes in pentanal, benzaldehyde, and furfurol were the most conspicuous in AS-ADKs. These volatile compounds, which are typically formed after drying, can be attributed to precursors in fresh samples that are degraded or reacted with one another during heating [51].

A similar trend appeared in Figure 6G,H, where the monomer and dimer of 3-pentanone presented a significant decline (*p* < 0.05) (Tables S1 and S2). In sharp contrast, the signal value of dimethyl ketone was markedly enhanced. The difference was that it tended to increase step by step in V0–V10, but tended to plateau in A0–A10. The dimer of 2-butanone also showed an apparent increase in the two methods. Evidently, dimethyl ketone presented the most notable change in ketones. This result may be attributed to the formation of aliphatic ketones via lipid degradation during hot-air drying [52].

As shown in Figure 6I, the contents of the monomer and dimer of 2-hexene-1-ol were dramatically higher in V0–V4 than in FKs. However, its trend demonstrated a steep drop in V6–V10. The peak value of the ethanol dimer exhibited a noteworthy decrease in V0. However, the content of the ethanol dimer in V1–V10 was close to that in FKs. After 10 h of drying, the content of α-terpinol increased, but the contents of 1-pentanol, the *Z*-3-hexene-1-ol monomer and dimer, and 1-hexanol were reduced. As shown in Figure 6J, the content of α-terpinol increased in A0–A10. Simultaneously, the other five alcohols presented a distinct downward trend, with the reduction of the ethanol dimer being the most prominent. The reason for this finding was probably that volatile alcohols were being primarily produced by ethanol dehydrogenase, and the increase in hot-air drying time led to the passivation or inactivation of relevant enzymes of synthetic aroma substances and caused the corresponding loss of volatile alcohol substances [53]. This result may also be attributed to the drying process promoting the esterification reaction of alcohols, reducing the alcohol content, and increasing the ester content [54].

### 2.5. Distinctive Features Analysis Based on PCA

PCA is normally used to reduce a set of original variables and to extract a small number of principal components in order to analyze relationships among the observed variables. PCA analysis showed that three different clusters are related at different sampling site positions at different periods [55]. Amorello et al. used a method similar to PCA—the linear discriminant analysis method—to identify almonds from different geographical origins [56]. The marinated pork hocks were clearly separated into three groups and nine odor-active compounds were determined to represent potential flavor markers for the discrimination of marinated pork hocks via Han et al.’s research results [57]. PCA proved that the volatile components of different tissue parts of tomato were distributed differently in the four cultivars [58]. In this study, similarly, the huge amounts of original data were initially subjected to PCA to reduce their dimensions. The integral peak area corresponding to each sample containing volatile organic compounds was normalized based on the z-score transform. All these normalized data were used to calculate the covariance matrix and its eigenvalues and eigenvectors. Then, the principal components were determined and the corresponding contribution rates were calculated. A classifying procedure was then used in a smaller space to illustrate the correlations between FKs and processed kumquats.

**Figure 7:**
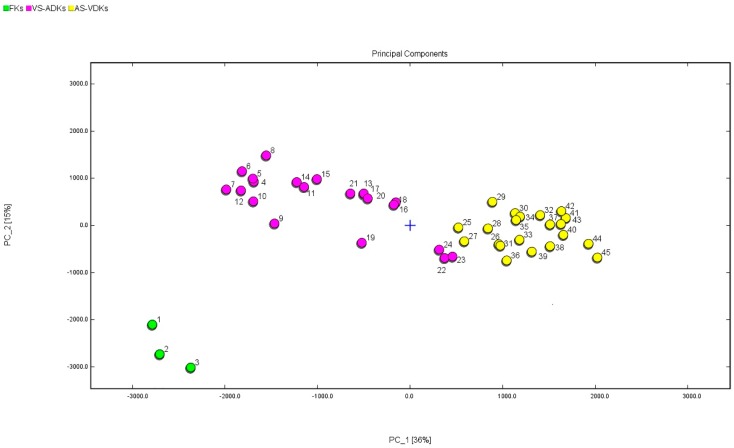
PCA based on the signal intensity obtained with different treatment processes of FKs, VS-ADKs, and AS-ADKs.

PCA was performed to highlight the dissimilarity in volatile profiles using signal intensities. The VOCs of FKs (1–3) evidently differ from those of processed kumquats (4–45) in Figure 7. The accumulative variance contribution rate of the first PC (36%) and the second PC (15%) was 51%, and the distribution map was presented via PCA. The results clearly showed that FKs, VS-ADKs, and APS-ADKs in a completely independent space could be well-distinguished in the visualization map. The FK samples could be confirmed by the negative score values of PC1 and PC2. Apart from the VS-ADK samples (22–24), the other VS-ADK samples (4–21) could be well-distinguished in accordance with the negative score values of PC1 and the positive scores of PC2. Meanwhile, AS-ADKs (25–45) can be well-defined on the basis of the positive scores of PC1 and can be separated by combining them with the scores of PC2. The flavor profiles of the two types of processed kumquats were clustered into various groups. The results showed that the data of HS–GC–IMS contained useful information that can be used as an effective means to differentiate among FKs, VS-ADKs, and AS-ADKs.

### 2.6. Clustering Analysis Based on the Heat Map

**Figure 8:**
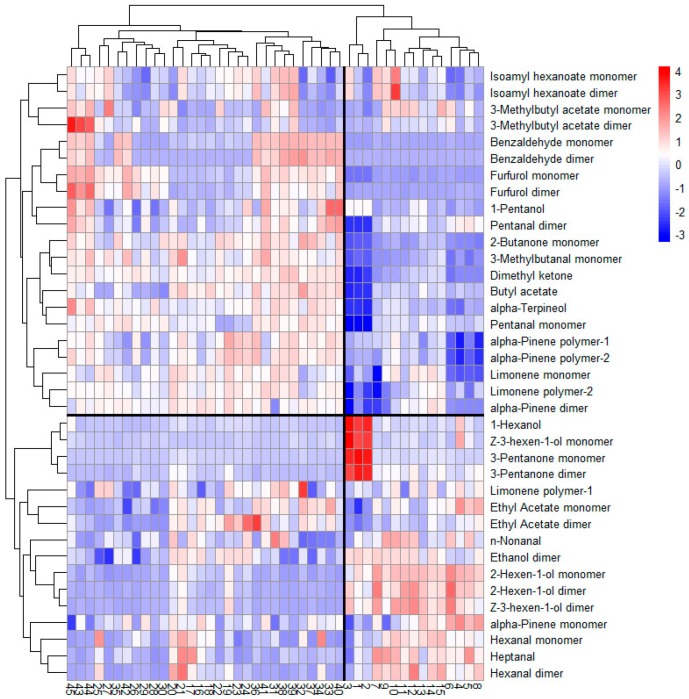
Heat map visualization and clustering results of the volatile constituents of different kumquat types.

To further understand the differences among FKs and the different processed kumquats, clustering analysis was conducted using the R programming method (Figure 8). Each variable was normalized on the basis of the z-score transform.

From the vertical mode, VS-ADKs (4, 5, 6 and 8), VS-ADKs (11–15), and VS-ADKs (7, 9 and 10) were clustered in three different forms. As the squared Euclidean distance increased, FKs (1–3) were grouped with VS-ADKs (4–15). The result showed that the volatile components of the preserved kumquats dried for 0–4 h after vacuum impregnation were highly similar to those of FKs. Therefore, the vacuum-impregnated and dried kumquats may preserve much of the similar compositions of FKs. This conclusion demonstrated that several components, such as the low contents of benzaldehyde and furfurol and the large amounts of 2-hexen-1-ol and *Z*-3-hexen-1-ol dimer, were basically the same. Similarly, the heat map illustrated that the similarity among VS-ADKs (43–45), VS-ADKs (25–30, 35 and 42), AS-ADKs (16–24), and VS-ADKs (31–34 and 36–41) was relatively high, such as 1-hexanol, the *Z*-3-hexen-1-ol monomer, and the 3-pentanone monomer and dimer. These samples were grouped together and then brought together with FKs (1–3) and VS-ADKs (4–15). On the basis of the cluster analysis, the differences among the processed kumquats were considerable in accordance with the cluster analysis results. This finding is consistent with the results of HS–GC–IMS and PCA. The differences could be primarily attributed to the formation of several aldehydes and the reduction of several alcohols.

From the horizontal mode, all the volatile components can be divided into two broad categories: one that tends to increase and one that tends to decrease overall. By analyzing the characteristics of the map from top to bottom, the amounts of isoamyl hexanoate, 3-methylbutyl acetate, benzaldehyde, furfurol, 1-pentanol, pentanal, 2-butanone, 3-methylbutanal, dimethyl ketone, butyl acetate, α-terpineol, α-pinene dimer and polymer, and limonene monomer and polymer-2 in AS-ADKs (16–24) and VS-ADKs (25–45) were determined to be increasing compared with those in FKs (1–3) and VS-ADKs (4–15). Evidently, these constituents were clustered. By contrast, the amounts of 1-hexanol, *Z*-3-hexen-1-ol, 3-pentanone, n-nonanal, ethanol dimer, 2-hexen-1-ol, α-pinene monomer, hexanal, and heptanal in AS-ADKs (16–24) and VS-ADKs (25–45) were decreasing compared with those in FKs (1–3) and VS-ADKs (4–15). Apparently, these constituents were also grouped together. Furthermore, 1-hexanol, the Z-3-hexen-1-ol monomer, and 3-pentanone were the most abundant compounds in FKs, while dimethyl ketone, butyl acetate, α-terpineol, and pentanal were the least abundant. The production of benzaldehyde and furfurol was positively correlated with the extension of the drying time in AS-ADKs, while the formation of the 2-hexen-1-ol and *Z*-3-hexen-1-ol dimer was negatively correlated with the extension of the drying time in VS-ADKs. These results are identical to those of HS–GC–IMS and PCA.

## 3. Material and Methods

### 3.1. Preparation of Materials and Candied Kumquat Fruits

**Figure 9:**
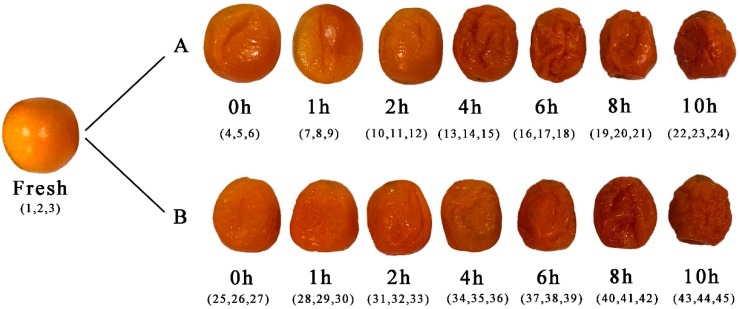
FKs, VS-ADKs (**A**), and AS-ADKs (**B**).

FKs with an initial moisture content of 83.79 ± 0.76% (g/g) were used. They were of the Guangxi variety and purchased from a local market in Hunan (China). Kumquats with a similar size and maturity were selected for the sugaring and drying experiments. The production of kumquat preserved fruits followed Chen’s method [59], with slight modifications. The steps included cleaning, blanching, hardening, cooling, cooking, sugar immersion, and drying. The sugar-soaking method involved dipping the fruits under atmospheric pressure and vacuum ambiance for 3 h. The drying method involved drying the fruits in hot air at 60 °C for 0, 1, 2, 4, 6, 8, and 10 h. After sample preparation, HS–GC–IMS was conducted for flavor determination. The schematic and sample numbers are shown in Figure 9.

### 3.2. Apparatuses

The following apparatuses were used in this study: vacuum-drying chamber DZF-2 (Yong Guang Ming Medical Instrument Co. Ltd., Beijing, China); DHG-9053A electro-thermostatic blast oven (Jing Hong Experimental Equipment Factory, Shanghai, China), and the HS–GC–IMS instrument Flavor Spec^®^ (the G.A.S. Department of Shandong Hai Neng Science Instrument Co., Ltd.; Shandong, China).

### 3.3. HS-GC-IMSAnalysis Methods

Preserved kumquat flesh (1 g) was weighed and transferred to a 20 mL headspace vial and then incubated in headspace volume at 40 °C for 20 min. Thereafter, 200 μL headspace was injected into the heated injector using a syringe in splitless mode at a temperature of 85 °C. Nitrogen (99.99% purity) was used as the carrier gas. Then, the samples were driven into an FS-SE-54-CB capillary column (15 m × 0.53 mm ID) by nitrogen at the following programmed flow: 2 mL/min held for 2 min, 15 mL/min maintained for 10 min, flow ramped up to 100 mL/min for 20 min, flow increased to 150 mL/min at 30 min, and flow then stopped. The analytes were separated at 40 °C in the column and then ionized in the IMS ionization chamber of 45 °C. Using this device, drift gas flow was set at a constant flow of 150 mL/min. All analyses were performed in triplicate. Volatile compounds were identified by comparing RI and the drift time standard in the GC–IMS library.

### 3.4. Data Analysis

The supporting analytical software included a Laboratory Analytical Viewer (LAV), three plug-ins, and a GC×IMS library search. It can analyze samples from different perspectives. IMS data were acquired and processed using LAV processing software. These data were used to view the analytical spectrum, where each point represented a VOC. Data were presented in a topographic map, where the *X*-axis denoted the ion migration time, the *Y*-axis denoted the retention time of the gas chromatograph, and the *Z*-axis denoted the peak intensity. Signal intensity was indicated by the color and can be used as a quantitative analysis tool.

The spectrogram differences between samples could be directly compared using the Reporter plug-in. The Gallery Plot plug-in can perform a fingerprint comparison to determine the differences in VOCs among different samples. Dynamic PCA, including sample clustering analysis and the rapid determination of unknown samples, was realized using MATLAB^®^ software. On the basis of the National Institute of Standards and Technology and IMS databases from the software’s built-in GC–IMS library search, a qualitative analysis of substances was achieved.

## 4. Conclusions

In this study, 40 signal peaks were identified from topographic plots in FKs and processed kumquats using HS–GC–IMS. All volatile compounds from FKs, VS-ADKs, and AS-ADKs belonged to five categories: terpene, ester, aldehyde, ketone, and alcohol. The variation tendencies of these compounds were affected by the different osmosis processing methods and these compounds increased in the drying period. Vacuum osmosis with sugar combined with hot-air drying was the most favorable for the formation of ethyl acetate in preserved kumquats. Osmosis with sugar at atmospheric pressure combined with hot-air drying was the most conducive to the production of benzaldehyde and furfurol. Ester led to the fruit flavor and floral aroma. It was a positive contribution to the good quality of preserved fruit. However, the aldehyde contributed to the roasted and burnt flavor of preserved fruit, which was a negative contribution to the good quality of preserved fruit. The flavor could be regarded as an important index to distinguish the quality of preserved fruit after processing. The volatile substances from vacuum-impregnated preserved kumquats were the most acceptable. Moreover, by using visualization methods, such as PCA and a heat map, the kumquats could be classified into three independent spaces and clustered into two separate groups. These analyses demonstrated that HS–GC–IMS is a reliable approach for determining and distinguishing the volatile profiles of different treated kumquats. Hence, the results are conducive to improving the understanding of volatile constituent release and the perception of preserved fruit consumption and to facilitating the efficient production of processed kumquats that meet consumer preferences.

## Figures and Tables

**Table 1:** Information of Qualitative Substances (40 peaks for 22 compounds).

No.	Compound	CAS#	Formula	MW ^a^	RI ^b^	Rt ^c^ [s]	Dt ^d^ [RIPrel]	Comment
1	α-Pinene	C80-56-8	C_10_H_16_	136.2	932.7	401.54	1.215	Monomer
2	α-Pinene	C80-56-8	C_10_H_16_	136.2	931.5	399.841	1.3022	Dimer
3	α-Pinene	C80-56-8	C_10_H_16_	136.2	932.7	401.54	1.6673	Polymer
4	α-Pinene	C80-56-8	C_10_H_16_	136.2	931.0	398.992	1.7309	Polymer
5	Hexanal	C66-25-1	C_6_H_12_O	100.2	795.9	252.139	1.2545	Monomer
6	Hexanal	C66-25-1	C_6_H_12_O	100.2	794.2	250.791	1.5622	Dimer
7	Heptanal	C111-71-7	C_7_H_14_O	114.2	899.0	354.588	1.3346	
8	2-Hexen-1-ol	C2305-21-7	C_6_H_12_O	100.2	850.5	301.544	1.1793	Monomer
9	2-Hexen-1-ol	C2305-21-7	C_6_H_12_O	100.2	849.3	300.288	1.5183	Dimer
10	Pentanal	C110-62-3	C_5_H_10_O	86.1	702.6	184.223	1.1766	Monomer
11	Pentanal	C110-62-3	C_5_H_10_O	86.1	700.7	183.013	1.4235	Dimer
12	Ethyl acetate	C141-78-6	C_4_H_8_O_2_	88.1	626.4	147.773	1.0936	Monomer
13	Ethyl acetate	C141-78-6	C_4_H_8_O_2_	88.1	625.2	147.254	1.3345	Dimer
14	3-Pentanone	C96-22-0	C_5_H_10_O	86.1	702.1	183.922	1.1085	Monomer
15	3-Pentanone	C96-22-0	C_5_H_10_O	86.1	702.1	183.922	1.3564	Dimer
16	Ethanol	C64-17-5	C_2_H_6_O	46.1	512.1	107.227	1.0469	Monomer
17	Ethanol	C64-17-5	C_2_H_6_O	46.1	518.2	109.088	1.1313	Dimer
18	Dimethyl ketone	C67-64-1	C_3_H_6_O	58.1	533.4	113.842	1.111	
19	Benzaldehyde	C100-52-7	C_7_H_6_O	106.1	957.2	439.544	1.1465	Monomer
20	Benzaldehyde	C100-52-7	C_7_H_6_O	106.1	955.0	435.993	1.4705	Dimer
21	α-Terpineol	C98-55-5	C_10_H_18_O	154.3	1166.6	864.748	1.2195	
22	*n*-Nonanal	C124-19-6	C_9_H_18_O	142.2	1103.6	729.481	1.484	
23	2-Butanone	C78-93-3	C_4_H_8_O	72.1	612.5	142.108	1.0562	Monomer
24	2-Butanone	C78-93-3	C_4_H_8_O	72.1	610.6	141.347	1.2482	Dimer
25	1-Pentanol	C71-41-0	C_5_H_12_O	88.1	766.2	228.27	1.2504	
26	Butyl acetate	C123-86-4	C_6_H_12_O_2_	116.2	811.9	265.683	1.2336	
27	Furfurol	C98-01-1	C_5_H_4_O_2_	96.1	829.7	281.606	1.0831	Monomer
28	Furfurol	C98-01-1	C_5_H_4_O_2_	96.1	828.6	280.624	1.3301	Dimer
29	3-Methylbutyl acetate	C123-92-2	C_7_H_14_O_2_	130.2	879.2	331.239	1.2998	Monomer
30	3-Methylbutyl acetate	C123-92-2	C_7_H_14_O_2_	130.2	879.0	330.987	1.741	Dimer
31	(*Z*)-3-hexen-1-ol	C928-96-1	C_6_H_12_O	100.2	855.7	306.615	1.228	Monomer
32	(*Z*)-3-hexen-1-ol	C928-96-1	C_6_H_12_O	100.2	855.5	306.47	1.5051	Dimer
33	1-Hexanol	C111-27-3	C_6_H_14_O	102.2	870.0	321.335	1.3242	
34	3-Methylbutanal	C590-86-3	C_5_H_10_O	86.1	666.2	165.191	1.1711	Monomer
35	3-Methylbutanal	C590-86-3	C_5_H_10_O	86.1	665.5	164.881	1.403	Dimer
36	Isoamyl hexanoate	C2198-61-0	C_11_H_22_O_2_	186.3	1217.2	991.484	1.5307	Monomer
37	Isoamyl hexanoate	C2198-61-0	C_11_H_22_O_2_	186.3	1217.5	992.272	2.1482	Dimer
38	Limonene	C138-86-3	C_10_H_16_	136.2	1029.4	568.854	1.2988	Dimer
39	Limonene	C138-86-3	C_10_H_16_	136.2	1028.0	566.046	1.6609	Polymer
40	Limonene	C138-86-3	C_10_H_16_	136.2	1027.6	565.344	1.7238	Polymer

^a^ MW: molecular mass; ^b^ RI: retention index; ^c^ Rt: retention time; ^d^ Dt: drift time.

## Supplementary Materials

The following are available online, Table S1: The Signal Values and Significant Differences of Five Volatile Components in VS-ADKs; Table S2: The Signal Values and Significant Differences of Five Volatile Components in AS-ADKs.

## Abbreviations

FKs Fresh	kumquats
VS-ADKs	Vacuum osmosis with sugar and then hot-air drying kumquats
AS-ADKs	Atmospheric pressure osmosis with sugar and then hot-air drying kumquats
PCA	Principal component analysis
HS–GC–IMS	Headspace gas chromatography–ion mobility spectrometry
VOCs	Volatile organic compounds
CAS	Chemical Abstracts Service
RI	Retention index
PCs	Principal components
