# Supplementary material for: Changes in the Volatile Components of Candied Kumquats in Different Processing Methodologies with Headspace–Gas Chromatography–Ion Mobility Spectrometry
Source: Molecules. 2019 Aug 22;24(17):3053. doi: 10.3390/molecules24173053 (PMC6749507; doi:10.3390/molecules24173053)
Supplement: Supplementary file 1 [file molecules-24-03053-s001.pdf]

Table S1 The Signal Values and Significant Differences of Five Volatile Components in VS-ADKs

Compound	FK	V0	V1	V2	V4	V6	V8	V10
Terpenes								
α-Pinene monomer	496.67±63.74 ^{c}	742.67±30.27 ^{a}	618.00±155.54 ^{abc}	601.00±34.60 ^{bc}	665.00±29.46 ^{ab}	645.00±40.85 ^{ab}	624.33±31.37 ^{abc}	559.67±53.21 ^{bc}
α-Pinene dimer	550.00±38.74 ^{c}	583.33±5.77 ^{b}	566.00±19.97 ^{bc}	634.00±12.29 ^{a}	656.67±9.02 ^{a}	662.67±4.51 ^{a}	665.67±7.77 ^{a}	653.33±7.51 ^{a}
α-Pinene polymer	760.33±13.32 ^{b}	678.00±37.69 ^{c}	736.00±79.90 ^{bc}	793.67±30.55 ^{ab}	765.33±38.59 ^{b}	789.67±21.73 ^{ab}	847.67±37.82 ^{a}	844.33±20.11 ^{a}
α-Pinene polymer	401.33±8.14 ^{cd}	343.67±14.74 ^{e}	387.00±52.51 ^{d}	430.67±15.18 ^{bcd}	417.67±21.50 ^{cd}	436.00±20.42 ^{bc}	466.00±21.93 ^{ab}	480.67±5.03 ^{a}
Limonene monomer	565.33±36.07 ^{b}	528.67±6.43 ^{b}	540.67±75.39 ^{b}	626.33±32.33 ^{a}	635.67±17.50 ^{a}	632.67±14.01 ^{a}	657.33±3.51 ^{a}	631.67±11.02 ^{a}
Limonene polymer	282.00±19.70 ^{ab}	315.00±21.93 ^{ab}	294.67±37.90 ^{ab}	295.00±69.28 ^{ab}	286.67±43.94 ^{ab}	286.67±43.82 ^{b}	330.00±27.62 ^{a}	291.67±39.93 ^{ab}
Limonene polymer	106.67±16.17 ^{d}	131.67±12.50 ^{c}	120.67±25.58 ^{cd}	142.67±8.14 ^{bc}	160.00±2.00 ^{ab}	155.67±6.66 ^{ab}	167.00±3.00 ^{a}	161.33±3.51 ^{ab}
Esters								
Ethyl acetate monomer	413.67±71.02 ^{c}	690.67±59.00 ^{a}	621.33±98.08 ^{ab}	575.00±68.94 ^{b}	566.67±44.77 ^{b}	611.33±38.14 ^{ab}	601.00±44.44 ^{ab}	506.67±20.65 ^{bc}
Ethyl acetate dimer	242.33±107.68 ^{d}	896.33±200.35 ^{bc}	846.67±369.44 ^{c}	753.00±161.69 ^{cd}	800.67±253.67 ^{c}	1080.00±269.51 ^{abc}	1417.67±323.62 ^{ab}	1607.33±494.55 ^{a}
Butyl acetate	19.67±1.53 ^{e}	77.33±4.93 ^{d}	79.33±4.51 ^{d}	86.00±4.00 ^{cd}	93.67±7.51 ^{bc}	114.33±6.81 ^{a}	116.67±8.33 ^{a}	100.33±6.81 ^{d}
3-Methylbutyl acetate monomer	59.33±29.30 ^{b}	150.33±57.06 ^{ab}	118.33±75.29 ^{ab}	212.67±6.43 ^{a}	162.00±66.70 ^{ab}	73.67±3.79 ^{ab}	136.33±59.68 ^{ab}	144.67±134.91 ^{ab}
3-Methylbutyl acetate dimer	15.00±2.00 ^{a}	26.00±12.00 ^{a}	24.00±14.93 ^{a}	23.33±2.08 ^{a}	25.67±4.51 ^{a}	15.33±1.15 ^{a}	19.00±8.54 ^{a}	25.33±3.21 ^{a}
Isoamyl hexanoate monomer	227.00±61.51 ^{ab}	179.67±35.22 ^{b}	262.67±50.50 ^{a}	280.00±60.63 ^{a}	232.33±30.44 ^{ab}	240.33±8.92 ^{ab}	249.33±48.95 ^{ab}	270.33±8.02 ^{a}
Isoamyl hexanoate dimer	31.33±15.63 ^{a}	21.00±7.81 ^{a}	40.33±17.67 ^{a}	46.00±26.00 ^{a}	32.00±5.29 ^{a}	32.67±4.04 ^{a}	35.00±14.80 ^{a}	39.00±3.61 ^{a}
Aldehydes								
Hexanal monomer	561.33±75.11 ^{bc}	656.00±15.10 ^{a}	633.67±47.61 ^{ab}	692.33±26.95 ^{a}	717.67±23.16 ^{a}	670.00±37.64 ^{a}	696.67±71.93 ^{a}	509.67±24.85 ^{c}
Hexanal dimer	476.00±201.17 ^{c}	786.00±99.51 ^{abc}	1062.67±46.80 ^{ab}	1213.67±276.33 ^{a}	1012.00±331.96 ^{ab}	657.33±188.62 ^{bc}	1217.33±379.75 ^{a}	440.33±88.49 ^{c}
Heptanal	127.33±34.53 ^{bc}	185.00±28.58 ^{ab}	202.00±26.96 ^{a}	187.67±14.64 ^{a}	162.33±28.22 ^{abc}	173.67±39.72 ^{ab}	178.33±45.83 ^{ab}	108.67±3.51 ^{c}
Pentanal monomer	37.67±15.31 ^{e}	447.33±10.97 ^{cd}	486.33±22.03 ^{abcd}	495.67±40.53 ^{abc}	534.33±28.22 ^{ab}	543.00±4.58 ^{a}	472.00±76.62 ^{bcd}	420.33±35.81 ^{d}
Pentanal dimer	13.33±2.52 ^{c}	1231.33±145.76 ^{a}	1025.67±200.89 ^{ab}	1018.67±108.86 ^{ab}	940.33±173.44 ^{ab}	869.00±224.46 ^{b}	911.67±168.52 ^{ab}	991.67±197.50 ^{ab}

Benzaldehyde monomer	50.00±8.66 ^{c}	99.33±20.21 ^{b}	116.00±8.72 ^{b}	104.67±14.01 ^{b}	99.33±7.51 ^{b}	234.67±32.33 ^{a}	223.67±29.02 ^{a}	206.00±26.00 ^{a}
Benzaldehyde dimer	11.67±1.53 ^{a}	13.00±2.00 ^{a}	14.67±1.53 ^{a}	13.33±0.58 ^{a}	11.00±2.65 ^{a}	11.00±2.64 ^{a}	13.67±3.79 ^{a}	13.33±1.15 ^{a}
n-Nonanal	76.00±19.67 ^{c}	104.33±16.77 ^{ab}	98.00±25.51 ^{ab}	119.33±7.09 ^{a}	75.67±10.41 ^{c}	89.67±15.63 ^{ab}	83.67±25.15 ^{ab}	80.00±21.93 ^{c}
Furfurol monomer	24.00±3.46 ^{d}	94.00±14.53 ^{c}	92.00±7.55 ^{c}	83.00±6.00 ^{c}	103.00±13.08 ^{c}	142.33±6.81 ^{b}	127.33±11.59 ^{b}	185.67±16.29 ^{a}
Furfurol dimer	12.67±1.53 ^{d}	18.00±1.73 ^{bc}	15.67±0.58 ^{cd}	13.67±3.06 ^{cd}	14.67±2.08 ^{cd}	20.33±3.21 ^{b}	17.67±0.58 ^{bc}	34.67±4.16 ^{a}
3-Methylbutanal dimer	36.33±8.02 ^{e}	63.67±17.62 ^{de}	79.33±2.31 ^{d}	91.33±9.71 ^{cd}	127.67±14.64 ^{bc}	145.00±14.53 ^{b}	194.33±52.73 ^{a}	157.67±18.88 ^{ab}
Ketones								
3-Pentanone monomer	687.33±25.42 ^{a}	47.67±9.02 ^{c}	57.67±16.56 ^{bc}	56.33±13.32 ^{bc}	79.00±16.00 ^{b}	51.00±12.53 ^{bc}	57.00±20.30 ^{bc}	40.67±2.89 ^{c}
3-Pentanone dimer	917.67±81.64 ^{a}	168.67±42.71 ^{bc}	260.00±25.24 ^{b}	266.00±69.16 ^{b}	210.00±103.18 ^{bc}	114.00±43.30 ^{c}	261.00±13.74 ^{b}	110.00±15.13 ^{c}
Dimethyl ketone	649.67±88.82 ^{f}	1390.33±108.65 ^{e}	1537.00±117.28 ^{e}	1871.67±162.81 ^{d}	2130.33±55.72 ^{c}	2337.00±53.25 ^{b}	2558.67±32.52 ^{a}	2675.33±38.48 ^{a}
2-Butanone dimer	20.67±4.51 ^{e}	63.67±4.73 ^{d}	65.00±18.08 ^{d}	77.67±13.20 ^{d}	111.00±5.29 ^{c}	130.67±2.89 ^{b}	185.67±8.08 ^{a}	176.67±15.14 ^{a}
Alcohols								
2-Hexen-1-ol monomer	342.33±82.56 ^{cd}	562.67±69.82 ^{a}	487.00±82.61 ^{abc}	504.33±9.50 ^{ab}	465.67±25.89 ^{abc}	367.67±30.92 ^{bcd}	225.67±44.00 ^{de}	188.00±39.23 ^{e}
2-Hexen-1-ol dimer	149.67±54.52 ^{ab}	272.33±84.76 ^{a}	224.33±95.14 ^{a}	266.33±56.90 ^{a}	197.00±19.00 ^{ab}	135.00±32.08 ^{ab}	45.33±19.09 ^{b}	42.67±10.02 ^{b}
Ethanol dimer	2283.67±79.00 ^{a}	1651.00±77.62 ^{b}	2265.67±102.91 ^{a}	2310.00±8.72 ^{a}	2057.00±256.73 ^{a}	2303.33±25.01 ^{a}	2025.33±286.64 ^{ab}	2261.67±19.60 ^{a}
α-Terpineol	17.00±3.00 ^{d}	48.67±13.61 ^{c}	71.67±11.24 ^{b}	78.00±9.54 ^{ab}	70.33±3.79 ^{b}	88.33±7.02 ^{a}	88.33±1.15 ^{a}	92.33±9.87 ^{a}
1-Pentanol	74.33±2.52 ^{a}	46.00±8.19 ^{c}	45.00±5.29 ^{c}	55.67±11.55 ^{abc}	58.67±12.90 ^{abc}	68.00±11.14 ^{ab}	51.67±7.37 ^{bc}	69.33±17.90 ^{ab}
Z-3-hexen-1-ol monomer	86.33±11.85 ^{a}	29.67±22.05 ^{b}	14.00±2.00 ^{b}	18.67±2.89 ^{b}	16.67±3.06 ^{b}	14.00±0.00 ^{b}	13.67±0.58 ^{b}	15.00±1.00 ^{b}
Z-3-hexen-1-ol dimer	112.33±37.58 ^{ab}	147.00±59.35 ^{ab}	110.00±58.13 ^{ab}	188.33±176.13 ^{a}	135.33±18.61 ^{ab}	98.67±30.99 ^{ab}	31.00±9.00 ^{b}	28.33±7.51 ^{b}
1-Hexanol	216.33±15.57 ^{a}	58.67±40.07 ^{b}	31.33±14.74 ^{bc}	29.67±6.43 ^{bc}	34.33±4.93 ^{bc}	35.33±4.16 ^{bc}	22.67±5.13 ^{c}	36.00±1.00 ^{bc}

Note: Different letters (a-e) indicate statistically significant differences between groups, $p < 0.05$.

Table S2 The Signal Values and Significant Differences of Five Volatile Components in AS-ADKs

Compound	FK	A0	A1	A2	A4	A6	A8	A10
Terpenes								
α-Pinene monomer	496.67±63.74 b	555.67±41.10 ab	599.33±62.00 ab	545.00±77.38 ab	573.33±35.80 ab	580.67±65.43 ab	659.00±14.73 a	500.67±115.34 b
α-Pinene dimer	550.00±38.74 b	649.00±6.00 a	640.00±2.65 a	629.33±39.12 a	648.67±6.66 a	649.00±8.00 a	658.00±2.00 a	639.67±16.62 a
α-Pinene polymer	760.33±13.32 b	810.33±26.39 ab	765.33±38.53 b	831.00±20.42 a	817.00±54.11 ab	846.00±8.89 a	781.00±54.44 ab	825.33±19.30 ab
α-Pinene polymer	401.33±8.14 c	446.00±18.03 ab	418.33±21.59 bc	467.67±4.93 a	459.67±34.31 a	468.33±10.97 a	429.67±30.89 abc	466.00±17.35 a
Limonene monomer	565.33±36.07 b	626.33±36.01 a	622.00±11.79 a	618.67±41.49 a	617.00±6.00 a	633.67±25.54 a	616.33±15.18 a	610.00±19.52 ab
Limonene polymer	282.00±19.70 ab	301.67±88.93 ab	280.33±24.58 ab	375.00±80.47 a	274.33±17.79 ab	292.33±93.36 ab	263.00±54.56 ab	251.00±59.73 b
Limonene polymer	106.67±16.17 b	157.00±7.81 a	152.67±4.93 a	157.00±14.93 a	161.00±3.00 a	160.00±10.15 a	162.67±7.02 a	159.00±2.00 a
Esters								
Ethyl acetate monomer	413.67±71.02 c	503.00±17.06 bc	464.33±62.01 c	679.00±66.05 a	611.33±70.51 ab	610.67±54.86 ab	528.00±150.12 bc	499.67±28.92 bc
Ethyl acetate dimer	242.33±107.68 c	383.33±9.61 bc	363.00±63.32 bc	1110.67±321.08 ab	858.67±142.68 a	978.00±291.36 abc	854.00±138.39 abc	770.67±206.78 abc
Butyl acetate	19.67±1.53 d	68.00±1.00 c	63.67±7.51 c	126.00±17.58 a	116.00±10.54 ab	118.00±1.00 ab	104.00±14.73 b	101.00±9.54 b
3-Methylbutyl acetatemonomer	59.33±29.30 a	144.67±134.91 a	129.33±57.74 a	101.67±68.06 a	79.00±35.37 a	153.00±75.62 a	141.00±79.27 a	215.67±24.03 a
3-Methylbutyl acetate dimer	15.00±2.00 a	34.33±4.04 a	20.67±11.59 a	20.33±14.57 a	14.67±3.06 a	28.00±15.72 a	21.67±0.58 a	80.33±8.08 a
Isoamyl hexanoate monomer	227.00±61.51 a	251.33±56.36 a	207.33±44.56 a	202.67±82.62 a	244.33±44.52 a	287.00±37.27 a	227.33±16.04 a	268.00±17.44 a
Isoamyl hexanoate dimer	31.33±15.63 a	36.33±15.53 a	26.00±8.54 a	27.33±17.04 a	36.00±15.72 a	45.67±16.20 a	28.67±6.81 a	41.33±6.43 a
Aldehydes								
Hexanal monomer	561.33±75.11 a	651.00±129.15 a	560.67±44.12 a	604.00±73.52 a	641.00±137.09 a	511.33±5.77 a	555.33±35.23 a	534.00±7.00 a
Hexanal dimer	476.00±201.17 a	511.00±403.17 a	259.00±61.94 a	320.33±75.04 a	471.33±342.94 a	229.67±18.15 a	279.33±27.15 a	285.00±31.76 a
Heptanal	127.33±34.53 a	127.00±22.11 a	110.67±8.50 a	122.00±9.17 a	136.00±6.93 a	130.00±6.56 a	129.33±17.90 a	118.67±6.66 a
Pentanal monomer	37.67±15.31 c	576.33±34.43 ab	584.33±17.79 ab	621.67±51.29 a	623.67±7.51 a	595.00±31.24 ab	609.33±33.50 a	547.67±23.76 b

Pentanal dimer	13.33±2.52 ^{d}	681.00±290.44 ^{c}	921.33±349.61 ^{bc}	1308±347.98 ^{ab}	1205±259.83 ^{ab}	1272±166.28 ^{ab}	1433.33±335.96 ^{a}	1494.33±162.86 ^{a}
Benzaldehyde monomer	50.00±8.66 ^{c}	180.67±95.52 ^{b}	99.67±9.29 ^{c}	716.33±51.81 ^{a}	709.67±3.06 ^{a}	740.00±18.52 ^{a}	703.67±26.50 ^{a}	673.67±27.30 ^{a}
Benzaldehyde dimer	11.67±1.53 ^{c}	15.00±6.24 ^{c}	11.67±3.06 ^{c}	330.67±65.31 ^{a}	299.33±1.53 ^{ab}	331.33±38.00 ^{a}	270.67±47.12 ^{ab}	257.00±41.58 ^{b}
n-Nonanal	76±19.67 ^{a}	61.67±26.50 ^{a}	76.00±17.35 ^{a}	72.00±58.97 ^{a}	58.33±18.61 ^{a}	77.33±39.11 ^{a}	77.67±18.45 ^{a}	65.00±20.07 ^{a}
Furfurol monomer	24.00±3.46 ^{e}	295.00±23.43 ^{c}	261.67±7.23 ^{d}	276.67±21.73 ^{cd}	283.00±12.49 ^{cd}	302.00±25.51 ^{c}	367.00±17.52 ^{b}	400.00±18.52 ^{a}
Furfurol dimer	12.67±1.53 ^{d}	78.33±14.84 ^{c}	64.33±14.98 ^{c}	57.00±8.89 ^{c}	64.67±7.23 ^{c}	73.67±11.85 ^{c}	104.00±8.89 ^{b}	145.33±17.01 ^{a}
3-Methylbutanal dimer	36.33±8.02 ^{d}	158.67±30.02 ^{bc}	144.33±26.03 ^{c}	163.00±12.17 ^{bc}	165.00±30.05 ^{bc}	173.33±8.96 ^{bc}	212.00±21.66 ^{a}	191.00±19.47 ^{ab}
Ketones								
3-Pentanone monomer	687.33±25.42 ^{a}	27.33±11.93 ^{b}	22.33±1.53 ^{b}	26.00±4.36 ^{b}	21.00±3.00 ^{b}	20.00±4.00 ^{b}	19.33±3.06 ^{b}	20.00±1.73 ^{b}
3-Pentanone dimer	917.67±81.64 ^{a}	95.33±61.78 ^{b}	88.67±13.58 ^{b}	82.33±3.79 ^{b}	70.67±24.42 ^{b}	72.33±5.03 ^{b}	75.67±18.82 ^{b}	68.67±10.50 ^{b}
Dimethyl ketone	649.67±88.82 ^{d}	2500.33±200.28 ^{bc}	2349.33±210.41 ^{c}	2602.67±106.10 ^{bc}	2577.00±123.84 ^{bc}	2900.67±143.39 ^{a}	2749.33±124.54 ^{ab}	2685.33±140.03 ^{ab}
2-Butanone dimer	20.67±4.51 ^{c}	116.67±12.01 ^{b}	144.67±37.54 ^{ab}	184.67±49.50 ^{a}	185.67±14.01 ^{a}	184.33±34.24 ^{a}	192.00±33.87 ^{a}	182.67±18.56 ^{a}
Alcohols								
2-Hexen-1-ol monomer	342.33±82.56 ^{a}	92.00±24.76 ^{b}	126.67±18.90 ^{b}	135.67±29.19 ^{b}	105.33±25.03 ^{b}	71.00±2.65 ^{b}	81.33±14.64 ^{b}	128.67±44.77 ^{b}
2-Hexen-1-ol dimer	149.67±54.52 ^{a}	13.33±3.21 ^{b}	17.33±1.53 ^{b}	21.00±8.19 ^{b}	16.67±4.62 ^{b}	13.33±2.52 ^{b}	15.00±3.61 ^{b}	22.00±7.94 ^{b}
Ethanol dimer	2283.67±79.00 ^{a}	596.67±126.46 ^{c}	1513.33±196.61 ^{abc}	1618.00±303.12 ^{abc}	1883.00±35.00 ^{ab}	942.67±43.00 ^{bc}	1729.33±142.58 ^{ab}	1636.33±177.71 ^{abc}
α-Terpineol	17.00±3.00 ^{c}	92.67±13.43 ^{b}	92.67±13.43 ^{b}	101.67±13.80 ^{ab}	105.33±5.13 ^{ab}	108.67±5.13 ^{ab}	91.67±3.06 ^{b}	120.00±19.00 ^{a}
1-Pentanol	74.33±2.52 ^{abc}	37.00±17.44 ^{c}	47.33±20.50 ^{bc}	84.67±37.75 ^{ab}	66.33±20.98 ^{abc}	74.33±8.96 ^{abc}	100.67±26.41 ^{a}	96.33±14.01 ^{a}
Z-3-hexen-1-ol monomer	86.33±11.85 ^{a}	12.67±0.58 ^{b}	14.33±2.08 ^{b}	15.00±2.65 ^{b}	13.67±2.52 ^{b}	14.33±2.31 ^{b}	13.00±1.00 ^{b}	15.33±2.52 ^{b}
Z-3-hexen-1-ol dimer	112.33±37.58 ^{a}	13.00±3.00 ^{b}	17.00±1.73 ^{b}	12.67±2.08 ^{b}	10.33±0.58 ^{b}	12.33±2.52 ^{b}	10.67±4.51 ^{b}	10.67±2.08 ^{b}
1-Hexanol	216.33±15.57 ^{a}	16.67±3.06 ^{b}	16.00±2.00 ^{b}	16.67±1.15 ^{b}	19.33±4.16 ^{b}	24.00±7.55 ^{b}	16.33±3.21 ^{b}	31.67±28.01 ^{b}

Note: Different letters (a-e) indicate statistically significant differences between groups, $p < 0.05$.
